# The grand challenge of autonomic disorders

**DOI:** 10.3389/fneur.2022.1052137

**Published:** 2022-11-01

**Authors:** William P. Cheshire

**Affiliations:** Mayo Clinic Florida, Jacksonville, FL, United States

**Keywords:** syncope, postural orthostatic tachycardia syndrome, autonomic neuropathy, orthostatic hypotension, multiple system atrophy, Parkinson disease, alpha-synucleinopathy, long COVID syndrome

The opportunities for discovery and improving treatments for patients with disorders of the autonomic nervous system have never been greater than now. Breakthroughs in autonomic neuroscience, connections with other neurologic subspecialties, and advances in medical technology have elevated classical autonomic medicine to an exciting new level. At the same time, autonomic medicine remains grounded in the essential clinical skills of listening to the patient, gathering a meaningful history, and performing a careful physical examination (1).

One finds in autonomic medicine the challenge of an unfolding—and underrecognized—crisis that is equally a timely opportunity to make a difference for patients and improve their quality of life. The crisis is the undersupply of autonomic expertise in proportion to need. Practitioners in the field recognize that autonomic disorders are extremely common and include syncope, orthostatic hypotension, postural tachycardia, diabetic autonomic neuropathy, and some forms of post-COVID syndrome, to name just a few. It is likely that any given person knows a family member or friend with an autonomic disorder. On the other hand, very little funding is currently available for research into autonomic disorders. If the levers of research funding are to turn, awareness of the relevance of autonomic disorders must increase. Part of the solution is the publication of cutting edge research and comprehensive review articles to advance and disseminate knowledge. Another part of the solution is an increase in the number of physicians, nurse practitioners, and other healthcare professionals who are knowledgeable about autonomic disorders to care for these patients.

## Classical autonomic medicine

In autonomic neurology one finds all the elements that tend to draw physicians to choose a career in neurology. Not only do disorders of the autonomic nervous system affect the function of peripheral nerves and ganglia in fascinating ways, but they display the remarkable complexity of the most interesting of organs—the brain—which distinguishes so much of what makes us human. Disruptions of autonomic function that may confuse or mystify others can be diagnosed by the experienced autonomic specialist who, like the fictional detective Sherlock Holmes, takes a careful history, is alert to subtle physical signs and, applying inductive reasoning to the art of clinical observation, reaches the hitherto elusive diagnosis (2, 3). Unlike Sherlock Holmes, the autonomic specialist also brings to the bedside the empathy of a physician or associated healthcare professional who, feeling compassion for the suffering, strives to translate science into solutions to provide understanding, hope, and healing.

To cultivate expertise in autonomic disorders is to specialize in generalization. Autonomic medicine combines the reductive methods of medical science with the holistic approach that regards physiology as an integrated whole and the patient as a person. In an age of increasing subspecialization, the autonomic specialist is a needed aberration. Even when the focus is on a single molecule, neurotransmitter, gene, or synapse, we know that such things are not single isolated phenomena but are keys to integrated systems that safeguard homeostasis and govern responses to stress. Because the autonomic nervous system penetrates every organ system and coordinates interrelated visceral functions, dysautonomias typically have systemic effects and require a collaborative multidisciplinary approach.

Sometimes an individual patient's diagnostic puzzle turns out to be a window to the next discovery in autonomic medicine. It may also instantiate important issues at the level of populations. The problems that prompt patients to seek autonomic consultation also signal opportunities for research in the quest to solve even larger questions.

## Autonomic medicine now

Building on the classical, cognitive approach to autonomic medicine, the specialty has achieved notable strides toward consistent terminology, indications for testing, and validated criteria for diagnosis. A number of notable developments in the field may be highlighted:

- One of the most interesting advances has been the identification of autoantibodies to the ganglionic acetylcholine receptor. Ganglionic blockade causes the clinical syndrome of autoimmune autonomic ganglionopathy, which presents with varying degrees of autonomic failure and is ultimately a condition that could be directly and objectively improved through immunomodulatory treatment (4).
- Genetic studies have elucidated the molecular basis for such disorders as familial dysautonomia, dopamine beta-hydroxylase deficiency, and familial orthostatic tachycardia due to norepinephrine transporter deficiency (5). Deciphering the fundamental basis of these rare disorders promises to provide further clues to the molecular mechanisms of more common disorders involving the same systems.
- The development of non-invasive clinical tests of autonomic function and their standardization have revolutionized the practice of autonomic medicine (6).
- Numerous studies have shown cardiac sympathetic neuroimaging to aid the differential diagnosis of Parkinson disease vs. multiple system atrophy. Although currently not reimbursed in the United States, third-party payers may reconsider once disease-modifying treatments become available and precise diagnosis, which is already important in defining prognosis, becomes even more actionable (7, 8).
- An important development has been the recognition of neurogenic orthostatic hypotension as a cardinal non-motor manifestation of alpha-synucleinopathies (multiple system atrophy, Parkinson disease, Lewy body dementia, and the Lewy body form of pure autonomic failure) as distinguished from other neurodegenerative disorders (for example, Alzheimer disease, progressive supranuclear palsy, and corticobasal degeneration) (9, 10).
- Within the past two decades, midodrine and droxidopa have become available for the treatment of neurogenic orthostatic hypotension, substantially improving the quality of life for many patients with autonomic failure (11).
- Recently, oligonucleotide-based disease-modifying drugs that promote degradation of mutant and wild-type ATTR mRNA have been approved for the treatment of transthyretin-related familial amyloid polyneuropathy, which can cause a severe autonomic neuropathy (12). The novel strategy of RNA-based therapeutics may hold promise for the future treatment of other neurologic diseases as well (13).

## Looking forward

The opportunities for further clinical research into autonomic medicine are too numerous to list. However, by pointing to a number of strategic areas, I hope to stimulate thought and catalyze ideas for potential research projects:

- Well-designed epidemiologic studies are needed to assess the prevalence of common autonomic disorders such as postural tachycardia syndrome (POTS). These should include objective data regarding symptom burden, economic burden, distribution across ethnicity and gender, longterm treatment outcomes, and recovery rate over time.
- There is an urgent need for the development of effective disease-modifying drugs for devastating autonomic disorders such as multiple system atrophy. Novel therapeutic approaches await discovery. The designs of prior negative trials nevertheless provide useful methodological frameworks once new candidate drugs are available to be tested.
- Among the non-motor manifestations of alpha-synucleinopathies, autonomic dysfunction increasingly has been recognized but remains insufficiently studied (14). Further research will be important to enable early diagnosis, accurate differential diagnosis, symptom management as disease progresses, and prevention of falls.
- Diabetes mellitus is the most common cause of peripheral neuropathy worldwide. Diabetic autonomic neuropathy, which affects ~20% of individuals with diabetes, can present with orthostatic hypotension, tachycardia, exercise intolerance, constipation, gastroparesis, sudomotor dysfunction, or erectile dysfunction. Diabetic cardiovascular autonomic neuropathy, in particular, is significantly associated with increased mortality and morbidity, including silent myocardial ischemia and stroke (15). Further research is needed into early detection, improved metabolic control, and pharmacologic therapies to ameliorate the symptoms and end-organ effects of autonomic neuropathy in people with diabetes.
- Supine hypertension and its adverse consequences have increasingly been recognized as a troublesome accompaniment of neurogenic orthostatic hypotension (16, 17). Further research is needed to guide clinical decisions about balancing the treatment of orthostatic hypotension with the potential to exacerbate supine hypertension in order to navigate between undertreatment and overtreatment.
- Whether interventions intended to treat orthostatic hypotension, if used over a long time, might increase the risk of developing hypertension is an understudied question. Midodrine and droxidopa, which are indicated for treating neurogenic orthostatic hypotension, are well-known to exacerbate supine hypertension, but their longterm efficacy and end-organ effects have not been well-studied (11). The potential longterm consequences of hydration with sodium chloride supplementation, which is considered first-line treatment for chronic orthostatic intolerance and posturally related syncope, are also largely unexplored (18). Evidence-based best practices for long-term therapy have yet to be well-defined.
- As data accumulate regarding the health effects of survivors of the COVID-19 pandemic, symptoms and signs of acute and chronic autonomic dysfunction are a topic of increasing interest. An estimated 2.5% of post-COVID patients have persistent symptoms of orthostatic intolerance (19). Further research is needed to elucidate the mechanisms of long-COVID fatigue and orthostatic intolerance, which may include direct tissue damage, cytokine storm, immune dysregulation, hormonal imbalance, or persistent low-grade infection (20), and to identify effective treatments.
- The detection of phosphorylated alpha-synuclein in cholinergic and adrenergic nerve fibers in the skin is a sentinel discovery in the search for an understanding of the pathophysiology of sympathetic neurodegeneration and holds promise as a potentially useful biomarker to diagnose Parkinson disease and multiple system atrophy (21–25).
- Gastroenterologic autonomic impairment is an important yet understudied area of autonomic medicine. Esophageal, gastric, and intestinal motility disturbances are frequently encountered in clinical practice, alone or in combination with other autonomic disturbances. More effective treatments are greatly needed.
- Another rich area for investigation is the role of the immune system in autonomic disorders, including sorting out which antibodies cause disease and might be targets for therapeutic intervention, and how to discern when antibodies are present incidentally without clinical consequence. Valid indications for intravenous immunoglobulin and objective criteria for assessing the response to treatment should be rigorously defined.
- The explosion of genetic information and the decreasing cost of some forms of genetic testing provide unprecedented opportunities to delve into the genetic and epigenetic influences on autonomic dysfunction including their vast implications for personalized medicine (26, 27).
- The appropriate role for wearable technologies such as smart watches in monitoring autonomic signs and assessing the results of therapy over time represent yet another frontier in autonomic medicine that has the potential to change medical practice profoundly. Distinguishing critical alerts from false alarms will continue to challenge patients and their physicians.
- Loss of thermoregulatory function can increase the risk of serious acute illness related to heat or cold exposure. Patients with global anhidrosis, for example, are at increased risk of potentially fatal heat stroke (28). The development of non-invasive methods for monitoring of core temperature during physical exercise or prolonged exposure to ambient heat or cold could save lives.
- The shift to telemedicine during the COVID-19 pandemic presents opportunities to define what optimal autonomic healthcare delivery to autonomic patients by virtual technologies should look like (29). The creation of multispecialty clinics and development of innovative care models for autonomic patients represent additional opportunities.
- Patients with autonomic disorders have needs that are not always met by current models of medical practice. Further research should examine these deficiencies and promote ways to improve the delivery of care to autonomic patients. Priorities include identifying barriers, discovering cost-effective models of care, and attaining more accurate diagnosis, better disease prevention, better symptom management, more individualized pharmacologic and non-pharmacologic therapies, and enhanced education. These needs exist not only in the developed world, but also in resource-poor regions.

## Looking collaterally

The English physician and physiologist John Newport Langley coined the term “autonomic nervous system” at the turn of the twentieth century (30). A pioneer in autonomic physiology, he systematically mapped out sympathetic and parasympathetic nerve functions in animal models using nicotine and pilocarpine. Langley obtained the acetylcholine receptor agonist nicotine from tobacco, which had been brought from Central America to Europe, where it attracted medical interest after Jean Nicot used it to cure the French queen Catherine of Medici's migraines. The first drug Langley studied was the muscarinic receptor agonist pilocarpine, which he acquired from the Brazilian plant *Pilocarpus jamborandi*. This interesting ethnopharmacological history illustrates how progress in autonomic medicine was then and continues to be truly a worldwide endeavor.

Successful progress in autonomic medicine requires a collaborative mindset and a robust exchange of ideas among the various specialties represented in the field, which include neurologists, cardiologists, internists, gastroenterologists, endocrinologists, and others. For the individual practitioner who comes across an interesting autonomic case or clinical question and would like to contribute to the field, unexpected opportunities for collaboration across specialties might exist just down the hallway or in the next building, particularly in academic centers.

Collaboration is also needed in the dissemination of new knowledge beyond the office of the specialist. Considering the attention that has been given to orthostatic hypotension, it is surprising how often measurement of standing blood pressure is omitted during physical examinations of patients who present with orthostatic symptoms. There is a pressing need for education of generalists and practitioners outside of the autonomic specialty. This includes not only the sharing of information but also the sharing of methods of education relevant to autonomic disorders.

Our patients are also our collaborators. We learn from them and seek to empower them to be active participants in managing their health. As we teach our patients, sharing our expertise with sensitivity and humility, there is still much for us to learn about how to explain their conditions clearly while correcting misinformation gleaned from the Internet. All too frequently, delays in diagnosis or misdiagnosis contribute to excessive worry or to a false perception of self-expertise as patients search for answers wherever they can, sometimes from unreliable sources. There is an enormous need for more high quality clinical research to provide a reliable foundation on which to base clinical judgment and to offer scientifically valid treatment recommendations. These areas are ripe opportunities for research and sharing of best practices.

## In conclusion

The field of autonomic disorders remains one of the most compelling fields in medicine. As our understanding of autonomic nervous system disorders continues to grow, much more remains to be done to meet the needs of patients (31). *Frontiers in Neurology* offers an exceptional opportunity for a more accessible, dynamic, and interactive exchange among the worldwide community of neurologists and experts in related and complementary fields who share an interest in autonomic disorders. Striving toward a common goal of excellence, together we can advance and share knowledge about autonomic disorders and make meaningful gains toward our ultimate goal of improving clinical outcomes and quality of life for our patients with autonomic disorders.

## Author contributions

The author confirms being the sole contributor of this work and has approved it for publication.

## Conflict of interest

The author declares that the research was conducted in the absence of any commercial or financial relationships that could be construed as a potential conflict of interest.

## Publisher's note

All claims expressed in this article are solely those of the authors and do not necessarily represent those of their affiliated organizations, or those of the publisher, the editors and the reviewers. Any product that may be evaluated in this article, or claim that may be made by its manufacturer, is not guaranteed or endorsed by the publisher.
